# Development of a Prediction Model for Short-Term Success of Functional Treatment of Class II Malocclusion

**DOI:** 10.3390/ijerph17124473

**Published:** 2020-06-22

**Authors:** Elisabetta Cretella Lombardo, Lorenzo Franchi, Giorgio Gastaldi, Veronica Giuntini, Roberta Lione, Paola Cozza, Chiara Pavoni

**Affiliations:** 1 Department of Clinical Sciences and Translational Medicine, University of Rome Tor Vergata, Via Cracovia, 50, 00133 Roma, Italy; robertalione@yahoo.it (R.L.); profpaolacozza@gmail.com (P.C.); dott.chiarapavoni@gmail.com (C.P.); 2 Department of Surgery and Translational Medicine, University of Florence, Piazza di San Marco, 4, 50121 Florence, Italy; lorenzo.franchi@unifi.it (L.F.); veronica.giuntini@unifi.it (V.G.); 3 Department of Orthodontics and Pediatric Dentistry, The University of Michigan, Ann Arbor, MI 48109, USA; 4 Dental School, Vita-Salute San Raffaele University, Via Olgettina Milano, 58, 20132 Milan, Italy; gastaldi.giorgio@hsr.it; 5 Department of Orthodontics, University Zoja e Këshillit të Mirë, Laprakë, Rruga Dritan Hoxha, 1000 Tirane, Albania

**Keywords:** Class II malocclusion, functional appliances, cephalometric analysis

## Abstract

(1) Background: The nature of the changes that contribute to Class II correction with functional appliances is still controversial. A broad variation in treatment responses has been reported. The purpose of this study was to find cephalometric predictors for individual patient responsiveness to twin-block treatment in patients with Class II Division 1 malocclusion; (2) Methods: The study was performed on a sample of 39 pubertal patients (21 females, 18 males) treated with the twin block appliance. Lateral cephalograms were available at the start of the treatment (T1) and at the end of functional therapy (T2). The outcome variable was the T2–T1 change in the sagittal position of the soft tissue pogonion with respect to the vertical line perpendicular to the Frankfort plane and passing through point subnasale. The predictive variables were age, gender at T1, and all the cephalometric parameters measured T1. Forward stepwise linear regression with *p* value to enter 0.05 and *p* value to leave 0.10 was applied; (3) Results: The only significant predictive variable that was selected was the Co–Go–Me angle (*p* = 0.000); (4) Conclusions: A greater advancement of the soft tissue chin on the profile is expected with smaller pretreatment values of Co–Go–Me angle.

## 1. Introduction

Several functional/orthopedic devices aimed at encouraging mandibular growth by the forward positioning of the mandible are available for the resolution of Class II division 1 malocclusion [1].

The twin block consists of two plates, upper and lower, which guide the mandible anteriorly by using interlocking occlusal bite blocks. The independent plates simplify language and eating with the device in place, improving patient compliance and treatment efficacy [2].

The nature of the variations that induce Class II resolution with functional appliances is not yet clear. Some authors suppose that the effects of functional therapy are limited to the dentoalveolar structures [3,4]. Other authors assume that this kind of device, applied during skeletal development, may modify maxilla–mandibular relationships [5,6].

In the literature, a series of previous studies have tried to find cephalometric predictors to produce a successful treatment [1,7,8].

Franchi et al. [1] found that a Class II patient at the pubertal growth spurt with a starting Co–Go–Me° smaller than 125.5° is expected to react successfully to treatment including Functional Jaw Orthopaedics (FJO). Similarly, Baccetti et al. [7] have suggested that Co–Go–Me angulation was predictive of both hard and soft tissue responses to headgear and Herbst appliance therapy. On the contrary, Fleming et al. [8] assessed that no correlation exists between mandibular morphology and vertical skeletal pattern, and favorable dento-alveolar and skeletal responses to twin-block treatment. Moreover, skeletal measurements—which include total mandibular length, ratio of posterior to anterior facial height, ramus height, overbite depth, cranial base length and occlusal predictors—have been related in different ways to an efficient treatment [9,10].

A large difference in treatment results has been reported, which may be due to the way in which differences in dentoskeletal patterns among individuals are handled in some studies. This variance can be overcome by accurate evaluations of preliminary data in the orthodontic diagnosis [11,12].

The aim of this research was to find cephalometric predictors for individual patient responsiveness to twin-block treatment in pubertal patients with Class II Division 1 malocclusion.

## 2. Materials and Methods

We followed the TRIPOD statement for transparent reporting of the multivariable prediction model for individual prognosis. This study was conceived as a Type 1a analysis. This study was approved by the Pediatric Ethics Committee of the Tuscan Region (01/2020) and by the Ethics Committee of the University of Rome Tor Vergata (201/16).

The research was carried out on a group of 39 pubertal subjects (21 females, 18 males) who were treated consecutively with the twin block device at two orthodontic clinics at the University of Florence and at the University of Rome Tor Vergata. All subjects treated by functional therapy at the peak in mandibular growth, as assessed by means of the cervical vertebral maturation method [13] (CS3–CS4 at the start of treatment (T1) and CS4–CS5 at the end of functional therapy (T2)), were included in this study.

Lateral cephalograms were available at T1 and at T2.

Treatment protocol consisted of a twin block device constructed following the design originally conceived by Clark [14]. The appliance was made up of maxillary and mandibular plates that fit against the teeth, alveolus, and other supporting structures. Delta or Adams clasps were constructed on both sides to anchor the upper plate to the first permanent molars, and 0.030-inch ball clasps (or arrow clasps) were positioned in the interproximal spaces anteriorly. The precise clasp arrangement depended on the state of the dentition at the moment of twin block construction. In the mandibular arch, Clark suggested placing ball hooks in the interproximal areas between the canines and incisors [14]. For all patients beginning twin-block therapy, the devices were realized from bite registrations taken with the incisors in an end-to-end position when the starting overjet was within 7–8 mm. If the initial overjet was greater than 7–8 mm, a two-step activation was carried out with the initial bite registration taken halfway between centric relation and incisal end-to-end position, with subsequent activation to edge-to-edge relationship 3–4 months later. Essentially, the construction bite was obtained to permit 5 to 7 mm of vertical opening in the area of the posterior bite blocks. An important advantage in the twin-block application is the opportunity to guide vertical eruption of posterior teeth through selective removal of acrylic throughout therapy. In hypodivergent subjects with a short lower anterior facial height and/or a deep curve of Spee, the acrylic on the posterior area of the upper bite block was trimmed to encourage the eruption of the lower posterior teeth [14]. All subjects included in the present research were recommended to wear the device full time (with the exception of meals and playing sports) until the end of therapy. The compliance with these instructions, however, varied among subjects.

The outcome variable was the T2–T1 change in the sagittal position of the soft tissue pogonion (Pg’) with respect to the vertical line (VL) perpendicular to the Frankfort Plane and passing through point Subnasal [7].

**Figure 1:**
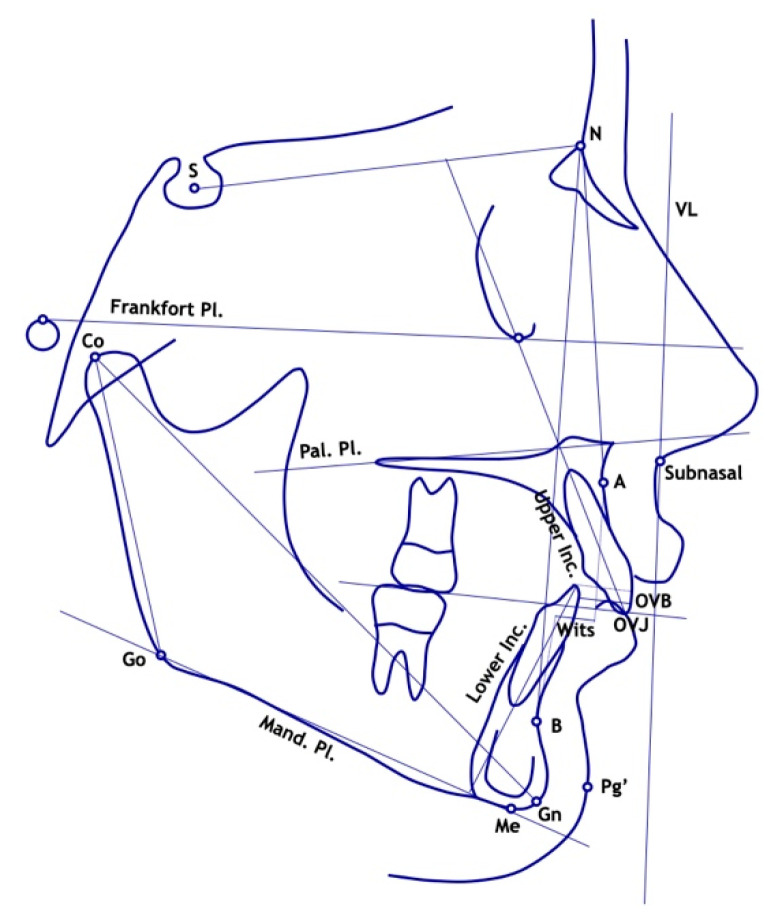
Cephalometric parameters measured at T1. (S = sella; N = nasion; Pal. = palatal; Pl. = plane; Mand. = mandibular; Inc. = incisor; OVJ = overjet; OVB = overbite; VL = vertical line; Go = gonion; Co = condilion; Me = menton; Gn = gnation; Pg’ = soft tissue pogonion; A = point A; B = point B).

The predictive variables were age and gender at T1, and the following cephalometric parameters measured at the start of treatment (T1). Sagittal skeletal relationships: SNA (°), SNB (°), Wits appraisal (mm). Vertical skeletal relationships: SN to palatal plane (°), SN to mandibular plane (°). Morphologic and dimensional mandibular measurements: Co–Go–Me (°), Co–Gn (mm), Co–Go (mm). Dental measurements: overjet (mm), overbite (mm), upper incisor to palatal plane (°), lower incisors to mandibular plane (°) (Figure 1).

All cephalograms were digitized and measured with cephalometric software (Viewbox version 3.0, dHAL Software, Kifissia, Greece).

### Statistical Analysis

Intra-rater reproducibility assessment for the cephalometric variables was performed on 15 randomly selected cephalograms after a 2-week washout period with Intraclass Correlation Coefficient (ICC, two-way mixed with absolute agreement). The random error was assessed with Springate’s method of moments estimator (MME) [15].

Descriptive statistics were used to summarize the demographics of the sample, the values of the cephalometric variables at T1 (predictors), and the T2–T1 change in Pg’–VL. The outcome variable was the T2–T1 change of Pg’–VL while the predictors were T2–T1 interval, age, gender, and the cephalometric variables at T1. Forward stepwise linear regression with *p* value to enter 0.05 and *p* value to leave 0.10 was applied. Variables found to be significant predictors (*p* < 0.10) were included in the multivariate model.

## 3. Results

The values for ICCs varied from 0.720 to 0.993, indicating substantial to almost perfect intra-rater agreement [16]. The MME random error measurements ranged from 0.3 to 1.0 degrees for the angular variables and from 0.3 to 0.8 mm for the linear measurements.

The average age of the sample was 11.7 ± 1.6 years at T1 and 13.5 ± 1.5 years at T2. Inclusion criteria consisted of an overjet greater than 5 mm, full Class II or end-to-end molar relationships, mandibular skeletal retrusion, and an improvement in facial profile when the lower jaw was postured in a forward position. The duration of comprehensive Class II treatment was 1.8 ± 0.6 years (min. 1.2 years–max. 2.4 years). The descriptive statistics for the cephalometric variables at T1 and for the T2–T1 changes for Pg’–VL are reported in Table 1.

The results for the forward stepwise linear regression are reported in Table 2 and Table 3. The only significant predictive variable that was selected was the Co–Go–Me angle (R square 0.563, *p* = 0.000). The prediction equation was Pg’-VL= −0.234*Co–Go–Me + 32.118. This means that a greater advancement of the soft tissue chin on the profile is expected with smaller pretreatment values of Co–Go–Me angle.

## 4. Discussion

The outcomes of this research showed that the Co–Go–Me angle is the single significant predictor for the amount of advancement of the chin after twin-block treatment for Class II malocclusion.

The studies performed by Petrovic and Stutzmann [17,18] have clearly shown that there is a positive relationship between mandibular growth potential and mandibular responsiveness to functional therapy.

Petrovic [19] proved that, in patients showing an anterior mandibular growth rotation, the responsiveness of the patients to FJO is significantly greater than in the subjects presenting a posterior mandibular growth rotation.

The angle formed by the condylar axis and the mandibular base is the main cephalometric expression of the morphological mandibular traits connected to anterior/posterior mandibular growth rotation [20,21]. The outcomes of this study are in agreement with other scientific papers [17], and they assert that there is a relationship between a small pretreatment mandibular angle and the evidence of an increased responsiveness to functional therapy.

In the present study, significant variations in the soft-tissue values were found after twin-block therapy. These results are similar to those reported by Morris et al. [22] and Lee et al. [23] in investigations on soft-tissue variations produced by twin-block therapy and by Flores-Mir and Major [24] in a systematic review on the same topic.

Greater improvement in the soft-tissue chin profile was obtained in Class II subjects who had a smaller Co–Go–Me angle at the start of the treatment.

From the outcomes of this study, it can be claimed that the presence of a mandibular retrusion with a small mandibular angle is the clinical indication for the application of twin-block therapy at puberty.

The results of the evaluations of starting features related to effective modifications in Class II subjects confirm previous data on the predictive role of mandibular morphology on favorable skeletal modifications produced by FJO at puberty [1,25]. Franchi et al. [1] suggested that greater increases in mandibular length in subjects treated with functional therapy are related to small starting values for the Co–Go–Me.

The present investigation revealed that, in these Class II subjects, the favorable mandibular skeletal change can also induce a favorable soft-tissue response at the chin.

This finding is also in agreement with Baccetti et al. [7] who reported 2.7 mm of relative forward movement of the pogonion in their prospective study involving two-phased treatment, which commenced with bonded Herbst therapy. However, in accordance with our study, Baccetti et al. [7] also found that the clinical indication for Class II treatment with functional therapy is a small mandibular angle (Co–Go–Me) and mandibular retrusion before treatment.

Contrary to our findings, in 2012 Fleming et al. [8] found that no relationship exists with respect to vertical dimensions and treatment outcomes with functional appliance therapy. In the retrospective study conducted by Fleming et al., [8] overjet reduction and changes in mandibular projection were positively correlated with the extent of the initial discrepancy. Moreover, forward movement of the chin during twin-block therapy was also found to be predicted on the initial overjet.

This study presents some limitations that have to be pointed out. The main limitation of the study is the increased possibility of bias because of the retrospective nature of the data collection. Another limitation is related to the short-term design of the present research, a longer observational period would give more information on the stability of the achieved results. Finally, the prediction model selected here should be validated on a different sample.

## 5. Conclusions

The main findings of this cephalometric study on the effects of twin-block treatment for Class II malocclusion were:

- Pretreatment of the Co–Go–Me angle was found to be the only significant predictor for the amount of advancement of the chin during twin-block therapy.
- A greater advancement of the soft-tissue chin on the profile is expected with smaller pretreatment values of the Co–Go–Me angle.

## Figures and Tables

**Table 1:** Descriptive statistics for the cephalometric variables at T1 and for the T2–T1 change for Pg’–VL.

Variable	Mean	SD	MIN	MAX
SNA	81.1	3.5	75.5	90.1
SNB	74.8	3.6	69.7	82.5
Wits	2.8	2.1	0.0	7.7
SN to Pal. Pl.	8.4	2.5	3.7	14.8
SN to Mand. Pl.	34.1	5.8	22.1	44.2
Co–Go–Me	124.1	6.3	113.2	141.6
Co–Gn	100.0	5.6	89.4	110.4
Co–Go	48.7	4.4	39.2	55.9
Overjet	7.1	1.9	2.6	10.6
Overbite	4.5	1.8	0.7	8.7
Upper Inc. to Pal. Pl.	112.2	7.4	89.9	132.6
Lower Inc to Mand. Pl	97.2	5.6	84.0	108.3
Pg’–VL	3.1	2.0	−0.9	6.8
T2-T1 Pg’–VL	1.9	0.5	1.0	2.8

(SD = standard deviation; Pal. = palatal; Pl. = plane; Mand. = mandibular; Inc. = incisor, MAX = maximum; MIN = minimum; T1 = start of treatment; T2 = the end of functional therapy).

**Table 2:** Forward stepwise linear regression analysis.

Table.	Estimate	Std Error	*p*-Value
Intercept	32.118	4.211	0.000
Co–Go–Me	−0.234	0.034	0.000

**Table 3:** Forward stepwise linear regression analysis: all other variables were not statistically significant.

Variable	t	Significance	Partial Correlation
T2-T1 interval	0.422	0.675	0.070
Sex	−0.994	0.327	−0.163
Age	−1.503	0.141	−0.243
SNA	0.887	0.381	0.146
SNB	0.549	0.586	0.091
Wits	−0.233	0.817	−0.039
SN to Pal. Pl.	−1.095	0.281	−0.179
SN to Mand. Pl.	−0.757	0.454	−0.125
Co-Go	0.698	0.490	0.116
Co-Gn	−0.267	0.791	−0.044
Overjet	1.672	0.103	0.268
Overbite	1.308	0.199	0.213
Upper Inc. to Pal. Pl.	−0.702	0.487	−0.116
Lower Inc. to Mand. Pl.	0.107	0.916	0.018

Pal. = palatal; Pl. = plane; Mand. = mandibular; Inc. = incisor.
